# On the Application of Vickers Micro Hardness Testing to Isotactic Polypropylene

**DOI:** 10.3390/polym14091804

**Published:** 2022-04-28

**Authors:** Hao Wu, Foram Dave, Mozaffar Mokhtari, Muhammad Mahmood Ali, Richard Sherlock, Alistair McIlhagger, David Tormey, Shaun McFadden

**Affiliations:** 1 School of Computing, Engineering and Intelligent Systems, Ulster University, Londonderry BT48 7JL, UK; 2 Department of Mechanical and Manufacturing Engineering, Centre for Precision Engineering, Materials and Manufacturing Research, Institute of Technology Sligo, F91 YW50 Sligo, Ireland; foram.dave@mail.itsligo.ie (F.D.); ali.muhammadmahmood@itsligo.ie (M.M.A.); tormey.david@itsligo.ie (D.T.); 3 National Graphene Institute and Department of Materials, School of Natural Sciences, The University of Manchester, Manchester M13 9PL, UK; mozaffar.mokhtari@manchester.ac.uk; 4 School of Engineering, Ulster University, Newtownabbey BT37 0QB, UK; a.mcilhagger@ulster.ac.uk; 5 Department of Life Science, Institute of Technology Sligo, F91 YW50 Sligo, Ireland; sherlock.richard@itsligo.ie

**Keywords:** isotactic polypropylene, Vickers hardness, carbon black addition

## Abstract

Hardness is a useful measure of a material’s resistance to permanent indentation; but for viscoelastic polymers, hardness data are highly dependent on the test type and the parameter set chosen. Vickers microhardness testing is used to leave small indents (<150 µm) and is shown to be applicable to polymers. A detailed investigation of the required steps for microhardness testing in isotactic polypropylene (iPP) is provided. Samples should be mounted in epoxy resin in order to maintain curing temperatures at room temperature. Mounted samples can be ground and polished in a semi-automatic polisher using graduated SiC paper (wet grinding) but progressing onto alumina suspension for polishing. Final polishing should be performed with 0.05-µm alumina suspension. The hardness measured was shown to be dependent on load and dwell time with a much greater dependency on dwell time. Strain recovery was shown to be completed after a time period equal to the dwell time. This study shows that indents can be measured thereafter, and it is recommended that they be measured within a 24 h period after the indent was created. After data fitting, the equation for hardness was shown to follow a power law with load and dwell time as the main variables. Fitting parameters were compared to those found in the literature, and it was found that parameters were significantly different to those reported elsewhere. Therefore, this study highlights the importance of calibrating on a case-by-case basis. Finally, to show the usefulness of the Vickers micro-hardness testing method, the calibrated test method was applied on iPP with additions of carbon black up to 3 wt.%. Comparisons were made with data from the literature, but the hardness data generated in our work were found to be at least twice that reported in the literature. The testing parameters were not cited in the literature: specifically, the dwell time was not provided, and this generated doubt on the usefulness of the cited data. Hence, this work is intended to serve as an exemplar of how to prepare and proceed with hardness testing of polymers.

## 1. Introduction

Hardness testing, defined as a test method to measure a material’s resistance to permanent indentation, was developed for metals but can be applied to polymers even though they exhibit a viscoelastic response that complicates the analysis [1,2]. However, it is only after appropriate calibration and parameter selection that hardness testing can be recommended for the measurement of permanent deformation of a polymer’s response to indentation [3].

Different types of hardness testing are available that can be classified against two basic defining aspects, namely, the loading procedure and the indenter geometry. Lopez [1] and Baltá Calleja [2] provide literature reviews of the common hardness testing methods used in polymer science. Hence, hardness numbers are specific to the hardness test method and indenter used. For metals, hardness numbers from different test methods are cross-referenced for equivalence against dedicated hardness scales [4]. On the other hand, due to their viscoelastic response and temperature response, defining equivalence between hardness scales for polymers is more complicated. Any attempt to cross reference polymer hardness numbers between scales is discouraged.

The first characteristic to consider in hardness testing is the loading-unloading procedure as this has a direct bearing on whether the measurement can be recorded ex situ (measured by inspection after the indenter is removed) or in situ (with the loaded indenter in place).

Typically, for ex situ measurement, the indenter is applied to the surface of the material until a target load is established and held constant for a prescribed time known as dwell time. The indenter is removed, and characteristic measurements are taken from the impression imparted on the surface. Generally, with this load-on, load-off procedure, the hardness number is obtained by dividing the load (kg) by the surface area of the impression (mm^2^). The surface area of the indent is estimated using the indenter geometry that made the indent.

In situ measurement of hardness is a rapid test procedure. In this case, the indenter is presented to the surface of the test specimen, and a minor load is applied to engage the point of the tool with the surface. Subsequently, a major load is applied for a specified time and the distance travelled, i.e., the depth achieved by the indenter into the material is the main measurement taken with the minor load reapplied. Typically, with this minor-major loading procedure, the hardness number is obtained from the distance travelled into the surface (i.e., the penetration depth) applied to the Rockwell equation for hardness.

As mentioned, the second characteristic for hardness testing is given by the geometry of the indenter. Brinell hardness testing, for example, uses a spherical indenter with a load-on, load-off procedure. One of the disadvantages of Brinell hardness testing arises due to the non-linear relationship between the area of the impression and the penetration depth; hence, to obtain reliable hardness values the impression diameter needs to be less than half and greater than a quarter of the indenter diameter. Vickers hardness testing uses a pyramidal indenter and a load-on, load-off procedure, which gives geometrically similar impressions regardless of the penetration depth. Two characteristic lengths are measured on the Vickers indent, the indent diagonals, and an average diagonal length is used to calculate the impression area. Load divided by area gives a Vickers hardness number. Knoop hardness testing is similar to Vickers, except that it uses an elongated pyramidal indenter. Rockwell hardness testing, on the other hand, uses the minor–major loading procedure and in situ measurement of depth. Two different indenter geometries are used in Rockwell testing: spherical and conical (also known as a Brale indenter). The Rockwell testing procedure is generally preferred for high volume production scenarios because it can give results more speedily than Vickers, Knoop or Brinell. For polymers testing, Rockwell test designations HRR, HRL, HRM, HRE and HRK (all using ball indenters) are applicable (ASTM D785 [5] and ISO2039-2 [6]). However, Rockwell testing is preferred for hard plastics where the results are less likely to be rate dependent.

A Barcol hardness (ASTM D2583 [7]) test is also suitable for rigid polymers (reinforced or otherwise). For softer polymers and elastomers, Shore hardness (ASTM D2240 [8]) (or Durometer hardness testing) is typical. An indenter is pressed into the material, and a reading is taken within a second of firm contact with the specimen. The hardness numbers read off instantly and are then referenced against a scale. Scales Shore A and Shore D are common with Shore A being used for softer polymers. The Barcol and Shore hardness tests are rapid methods useful for quality assurance purposes. They are not suitable for precision scientific work due to measurement uncertainties associated with manual operation.

A further distinction given on the Vickers test is known as micro Vickers hardness testing. In this case, the indenter load is kept below 2 kg, and generally, the diagonals are measured using an optical microscope due to the small length scales involved. Micro Vickers testing gives hardness at precise locations and is therefore used frequently in scientific laboratories. Because the indents are measured at the microscale, sample preparation, involving grinding and polishing, must be used, as standard, prior to the indentation in order to achieve reliable results.

Due to their viscoelastic response, polymers are also tested using the Wallace hardness procedure. The Wallace test uses a Vickers indenter but with a minor-major loading procedure. The travel distance of the indenter is monitored and recorded continuously even after the major load is removed to give deflection versus time and the viscoelastic response during load and recovery. Penetration depth is the measured quantity; however, indenter geometry is used to estimate the diagonal length of the impression used in the standard Vickers hardness equation (introduced in the next section) to give a hardness number. Although the Vickers indenter and the Vickers equation are used in the Wallace test, care should be taken not to equate a peak Wallace hardness number directly with a traditional Vickers hardness test number. The loading and measurement procedures are very different, and this means that classic Vickers testing cannot capture load recovery information to the same extent that the Wallace test can. This feature was highlighted by Suwanprateeb [9] who presented hardness data comparing diagonal length (ex situ) versus penetration depth (in situ) measurement methods for HDPE and PMMA polymers. Differences in the calculated Vickers hardness numbers between the alternative methods were as high as 60% at 1 kg load and dwell times of 1 s. The differences between the hardness calculations between methods became insignificant as the load was increased to 5 kg for PMMA and 2 kg for HDPE. In addition, the in situ depth penetration displayed a load dependence as well as a dwell time dependence. Correspondingly, the ex situ diagonal length measurement method for hardness showed time dependence only, that is, the hardness measured as a function of diagonal length was load independent. Overall, the data presented in Reference [9] highlights the challenge faced when using hardness as an absolute measure of a material property, especially. Similarly, Parashar et al. [10] studied the relationships between the different load levels (varied from 5 g to 160 g) and the Vickers hardness of PETP, PTFE and PP. They used a fixed dwell time of 30 s throughout. The results showed that the hardness measurements of PP sharply increased from 5 g to 10 g and remained at stable levels as the loads were over 20 g. The behaviour at very low loads was attributed to the depth of the indenter being comparable to the depth of the distortion zone where any further loading would cause intense slipping of polymer chains. Hardness in polymers is highly dependent on test parameters selection, and this prohibits casual cross referencing of hardness numbers not just between different hardness scales but even within the Vickers scale itself. Vickers hardness numbers in polymers need to be cited along with the load level, the dwell time and the measurement method (i.e., penetration depth or diagonal length).

Crawford [3] performed a dedicated study of microhardness testing, whereby Wallace testing was compared with traditional Vickers testing. After the differences were highlighted, Crawford showed that, with a careful selection of test parameters, meaningful and repeatable hardness data can be acquired from the traditional Vickers test even though the polymers tested displayed viscoelastic responses. It was discovered that once the indenter is removed the depth recovers significantly (by 50–70%) because of the viscoelastic response. Importantly, however, the corresponding recovery on the diagonals was less than 5% [3]. This latter point of low recovery of the diagonals makes traditional hardness testing feasible for polymers.

Another hardness measurement approach is called Depth Sensing Indentation (DSI) or nanoindentation, which uses a Berkovich indenter and the Oliver–Pharr load–unload method to give hardness and modulus [11]. Although its application to polymers is somewhat controversial, Giró-Paloma et al. [12] provided a framework for DSI applied to polymers. However, this work focuses on the suitability of micro Vickers hardness of isotactic polypropylene (iPP) and offers a framework for comparing hardness data reported in the literature that used alternative testing parameters. Polypropylene is a semi-crystalline polymer where the degree of crystallinity is dependent on parameters such as tacticity, fillers type, concentration and processing conditions. Isotactic polypropylene is polymorphic and can have three crystalline modifications including: α, β and γ forms. Isotactic polypropylene crystallizes into α-form under traditional processing conditions; β-modification is formed under special processing conditions or in the presence of nucleating agents, and γ modification is found in low-molecular weight iPP and random copolymer polypropylene [13,14]. Both the degree of crystallinity and the proportion of the crystalline modifications affect mechanical properties. Martinez Salazar et al. [15] investigated Polyethylene (PE)-polypropylene (PP) blends via microhardness testing but in so doing needed to characterise the microhardness of PP independently from PE. They showed that an additive law described based on crystallinity was suitable to describe the microhardness of PP as follows:

(1) $${HV=\alpha H}_{C\;}+(1\;-{\;\alpha )H}_{A},$$

where α is the percentage crystallinity; *H*_C_ is the hardness of fully crystalline PP, and *H*_A_ is the hardness of fully amorphous PP. Using hardness testing with loads of 0.25 and 0.5 N and with a loading cycle of 0.1 min, Martinez Salazar et al. were able to extrapolate to show *H*_C_ = 30 MPa and *H*_A_ = 116 MPa. Whereas, this is useful information, there is ambiguity with regards to the hardness testing parameters. It was not clear which load was used consistently (i.e., 0.25 or 0.5 N). Furthermore, the cycle time was 0.1 min or 6 s. Crawford’s work shows that hardness values are strongly dependent on dwell time; therefore, great caution must be exercised when cross referencing the hardness data in Reference [15] against hardness data obtained over longer dwell periods (as recommended by Crawford).

A clear example of the difficulty in cross referencing hardness data from different sources was outlined by Flores et al. [16]. They investigated the microhardness of polymer-carbon black (CB) composites at different levels of CB addition. They cited data from Koszkul [17] who provided hardness data on the effect of CB addition into iPP, but the starting data, i.e., iPP without CB addition, showed microhardness of 29 MPa with α of around 0.5. This value was seen as too low and could not have been reconciled with the data presented in other sources since no dwell period for the hardness test was cited in the original information source. Hence, the difficulty with cross referencing hardness data without full knowledge of the testing parameters was demonstrated. Seidler and Koch [18] investigated the relationship between different heat treatment temperatures and the Vickers hardness of α- and β-crystalline phases iPP. They also reported different creep tendencies and elastic moduli of the two types of iPP using Depth Sensing Indentation (DSI) or nanoindentation. However, this study only chose a single set of testing parameters with a load of 1 g and a dwell time of 30 s for Vickers hardness testing.

In order to analyse polymers for hardness testing, much care and attention must be given to the sample preparation using typical mounting and polishing procedures. However, there is little detail of sample preparation found in the literature, which represents a significant gap. The determination of the testing procedure (including sample preparation) and parameters is of paramount importance so that meaningful results can be determined.

The aim of this study is to determine the suitability of micro Vickers hardness testing to iPP. In particular, the research question is what empirical and mathematical framework, if any, must be adopted to allow for comparisons to be made between different hardness datasets obtained under different test parameters? The study will investigate the appropriate steps to take in terms of sample preparation, so that repeatable and reliable results can be achieved. Detailed objectives include:

1. Investigation of mounting iPP samples in other cold-casting polymers such that the curing temperature is kept below 25 °C.
2. Establishment of suitable grinding and polishing processes for mounted iPP samples.
3. Investigation of the relationship between hardness and dwell time at different load levels and, through data fitting, finding empirical data for iPP regression equations.
4. Development and validation of hardness protocols for isotactic polymer at low load levels between 5 and 50 g.

The relevance of the final objective is made clear by considering that lower load levels lead to smaller indents with smaller diagonals (less than 150 µm). Many modern Vickers hardness testers are limited in the size of indent that they can measure (indents on metals are typically less than 150 µm); hence, lower loads facilitate the usage of a greater number of modern hardness testers. However, this work avoids the very low load (<5 g) regimes shown by Parashar et al. [10] where hardness increases at a high rate with load increase due to the indenter depth being comparable to the size of the distortion zone. In the proposed experimental loading regime, it is expected that the indenter depth will penetrate to levels where the effect of the distortion zone in advanced of the indenter diminishes because the penetration depth is larger than the extent of the distortion zone itself (according to ref. [10]).

Finally, to demonstrate the applicability of the hardness testing method, a series of tests will be performed on iPP with varying wt.% of CB. The purpose of testing with carbon black addition is to demonstrate hardness testing as a comparative, heuristic test method suitable for quality control.

## 2. Materials and Methods

### 2.1. Sample Manufacture

**Figure 1:**
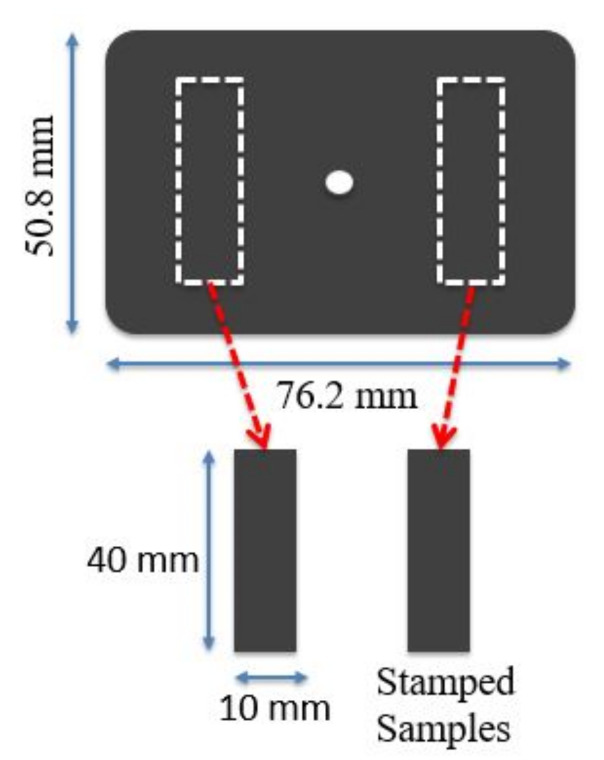
Schematic representation of the moulded coupons indicating the locations used to create stamped sample strips.

The iPP samples for testing were fabricated from a poly(propylene-co-ethylene) base resin (CAS 9010-79-1). The polymer has a specified density of 0.90 g/cm^3^ and a melt flow rate (MFR) of 25 g/10 min. The resin was compounded with small amounts (<2 wt.% total) of additives that assist the injection-moulding process. The compounded material was then injection moulded into coupons of 76.2 × 50.8 × 2.0 mm^3^. A maximum moulding temperature of 216 °C was employed with an injection pressure not exceeding 10 MPa. Sample strips 40 × 10 × 2 mm^3^ were stamped out of the coupons as indicated in Figure 1. This was done to eliminate any material variation that may arise from modified material flow close to the edges during the injection moulding process.

Further sample sets containing mesoporous carbon black (CB) (CAS 1333-86-4) were fabricated in a similar manner with 0.5, 1, 2 and 3 wt.% CB being added during the compounding step.

### 2.2. Hardness Testing

Standard sample preparation using a QATM Saphir 520 semi-automatic polisher (ATM Qness GmbH, Mammelzen, Germany) consists of mounting, grinding and polishing of the intended surface for testing. An objective of sample preparation is to ensure that the results are unaffected by the sample preparation processes. For example, mounting may consist of raising the temperature of the sample which could affect the mechanical properties of the sample under investigation. Hence, a requirement set out in this investigation was to ensure that the temperature does not exceed 25 °C. Details of the sample preparation are described herein.

#### 2.2.1. Equations

Vickers hardness is given by the number:

(2) $$HV=2sin68{}^{\circ}\left(\frac{F}{D^{2}}\right)=1.8544\left(\frac{F}{D^{2}}\right),$$

where *F* is load in kg, and *D* is the average length of diagonal in mm. An important measurement parameter known as the reduced time was defined as the ratio of time after unloading to the time that the load was applied [3]. Essentially a reduced time of unity (as a minimum) was recommended, which means that the waiting time after load removal should be at least equal to the dwell time before measurements are taken. After a reduced time ratio of unity, the recovered strain was observed to be unchanging, and the diagonal measurements were assumed as a permanent feature of the indent.

To develop the analysis, Crawford [3] assumed that the load–diagonal length relationship followed the expression:

(3) $${F=a\;D}^{n},$$

where *a* and *n* are fitting constants. Furthermore, the parameter *a* was also found through least squares to fit an expression of the form:

(4) $${a=A\;t}^{B},$$

where *A* and *B* are fitting constants, and *t* is dwell time.

By substituting, Hardness values are shown to be a function of load and dwell time as follows:

(5) $$HV=1.8544{\;F}^{(n-2)/n}{\left({At}^{B}\right)}^{2/n}.$$

For practical purposes, Crawford [3] simplified the equation for polymers hardness to

(6) $${HV=K\;F}^{x}t^{y}.$$

The indices *x* and *y* can be related to the indices in Equation (5). Similarly, the constant can be seen to be equivalent to *K* = 1.8544*A*^2/n^.

#### 2.2.2. Mounting

Samples were cut lengthwise to fit inside moulds of diameter 40 mm and depth 30 mm. Samples were placed on the mould floor with their longest, narrow faces directed downwards. Household adhesive (cyanoacrylate) was used sparingly to adhere the samples, which are buoyant in the mounting compounds, to the floor surface of the mould.

Several cold casting compounds that do not require the action of external heat were considered. The three cold mounting compounds investigated were two epoxy resins (Epo-set^TM^ (MetPrep, Coventry, UK) and Epofix^TM^ (Struers, Cleveland, OH, USA)) and one acrylic-based compound (Vari-set^TM^ (MetPrep, Coventry, UK)). The typical curing times for each compound are 5 h for Epo-set^TM^, 12 h for Epofix^TM^ and 0.5 h for Vari-set^TM^.

Even though the mounting compounds are considered as cold processes, the curing processes for these compounds are intrinsically exothermic. Hence, it was important to monitor the temperature of each compound as it cured. A type-K thermocouple was inserted into each mounting compound to record the temperature history during the curing process.

#### 2.2.3. Grinding and Polishing

The sample surfaces were prepared by wet grinding and polishing through graduated steps. Silicon carbide paper was used initially to grind to the target surface using P400 grade. Subsequently, the surface was ground using a complementary rotation regime with grinding papers P800, P1200 and P2500 each under a load of 15 N and a rotation speed of 200 rpm for 2 min at each grit rating. The polishing steps were performed in a graduated manner using 5, 1, 0.3 and 0.05 µm alumina suspensions on a clean napless cloth. Each polishing stage was conducted under a load of 15 N and a rotation speed of 150 rpm for 3 min each using a contra-rotation regime. The surface quality was frequently monitored by viewing under a microscope in-between grinding and polishing stages to ensure that deeper scratches from the previous preparation stage were removed.

### 2.3. Hardness Measurement

A Future-tech FM-800 microhardness tester (Future-tech, Kawasaki, Japan) was used in this study. Day-of-use calibration tests on a standard metal test block were performed prior to all measurements. Four load levels (5, 10, 25 and 50 g) were selected with each tested with seven levels of dwell time (5, 15, 30, 60, 120, 240 and 360 s).

Pure (neat) iPP samples were used throughout the calibration procedure. In order to observe the viscoelastic response and to ensure the permanency of indent, a series of tests were devised whereby the diagonals were measured regularly at timed intervals after load removal. This measurement was performed down the eyepiece, and to expedite the process, only one diagonal was measured for each indent. The delayed ratio (also known as the reduced time) is defined as the ratio of the delayed time of measurement to the dwell time. A delayed ratio of 0.5 for 360 s of dwell time would mean that the measurement of the diagonal took place 180 s after the indent was made. However, it should be noted that a ratio of 0 would not truly represent the point of load removal since the retreat of the indenter and switch between the indenter and the object lens could take around 10 s. Nevertheless, the purpose of this initial test was to ensure that a stable, reliable and permanent indent measurement could be achieved. Each indent was measured again after a rest period of 24 h to see if any significant change or recovery of the diagonal measurements had occurred over that time period. It has also been demonstrated elsewhere that hardness values measured 72 h after load removal are very close to the results measured right after the indentation [19]. Reliable measurement of indents after a rest period of 24 h is preferable for practical reasons as this would facilitate laying numerous indents on one day with the arduous task of measuring diagonals over the next day.

Once the rest period was established, indent measurement was performed using a Leica DMi8 optical microscope (Leica Microsystems, Wetzlar, Germany). The image of the indent was captured with 100× or 50× magnification in bright field mode with a polarisation applied to better identify the edges of the indent. Vickers hardness value was then calculated based on the average size of the diagonals of the indent using the expression in Equation (2).

### 2.4. Data Fitting

According to Equation (3), load, *F*, plotted against diagonals, *D*, on a log–log scale will give a linear trend with a slope equal to the index *n*. According to Equation (4), if dwell time, *t*, is varied as a parameter in the data, then the constant *a* from Equation (3) can be plotted against dwell time on a semi-log plot to give another straight-line relationship. From this linear relationship, it is possible to determine the parameters *A* and *B* of Equation (4) from the y-intercept and the slope, respectively. Once the experimental data are plotted as just described, regression analyses can be used to give the fitting parameters leading to the full expression in Equations (5) and (6). Since each load case will be tested at different dwell times, multiple trendlines of load versus diagonals will be available. In this case, the average slope, $\bar{n}$, will be deduced from the family of trendlines.

## 3. Results

### 3.1. Mounting Compound Temperatures

**Figure 2:**
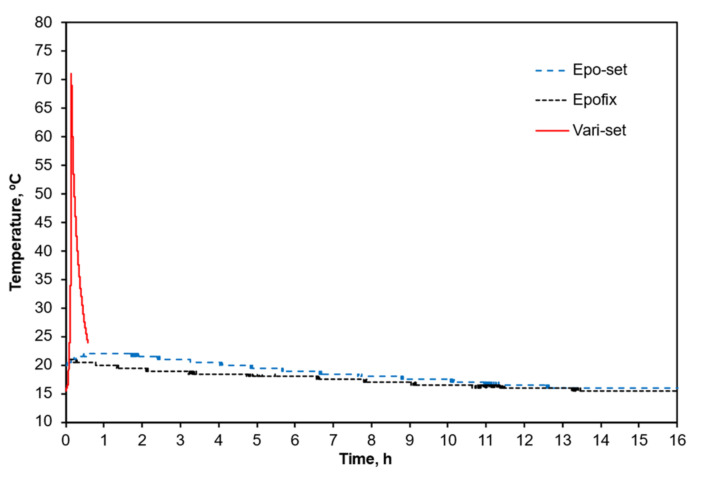
Comparisons of the temperature histories during curing of Epo-set^TM^, Epofix^TM^ and Vari-set^TM^ mounting compounds.

Figure 2 shows the temperature data acquired from the curing processes involving all three mounting compounds. As expected, the acrylic-based compound (Vari-set^TM^) exhibited the highest exothermic thermal response with a peak temperature of around 70 °C and cured the fastest (within an hour). The epoxy resin compounds stayed below the threshold temperature of 25 °C but required longer curing times (each fully cured after approximately 12 h).

### 3.2. Sample Preparation and Indent Observation

**Figure 3:**
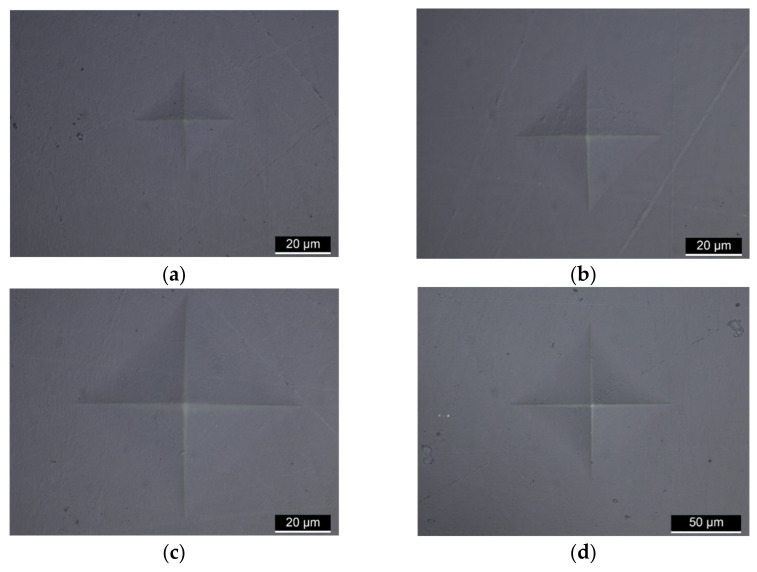
Optical microscope images of the (**a**) 5 g, (**b**) 10 g, (**c**) 25 g and (**d**) 50 g indents (**a**–**c**) taken at 100× magnification; indent (**d**) at 50×. Indent (**d**) is the largest (note the scale bar).

Following on from the mounting processes, the grinding and polishing stages gave an acceptable surface finish when viewed under the microscope. Figure 3 shows examples of indents left after the various loads. Using the brightfield with polarisation, the corners of the indent (the cruciform shape) are clearly visible after indentation. Importantly, there is no evidence of embedded polishing particles as can sometimes be apparent after applying a suspension medium to soft materials. Indents at all load levels are shown and it is clear that the load at 50 g gave the largest indent such that the magnification level had to be reduced to include the indent within the field of view.

### 3.3. Strain Recovery

**Figure 4:**
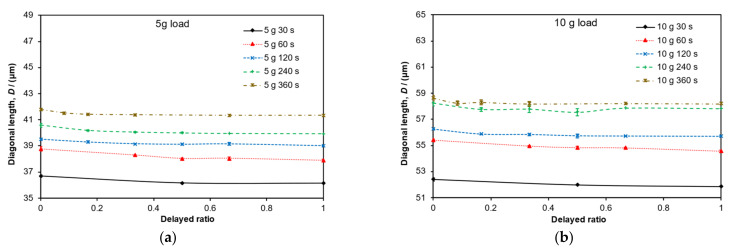

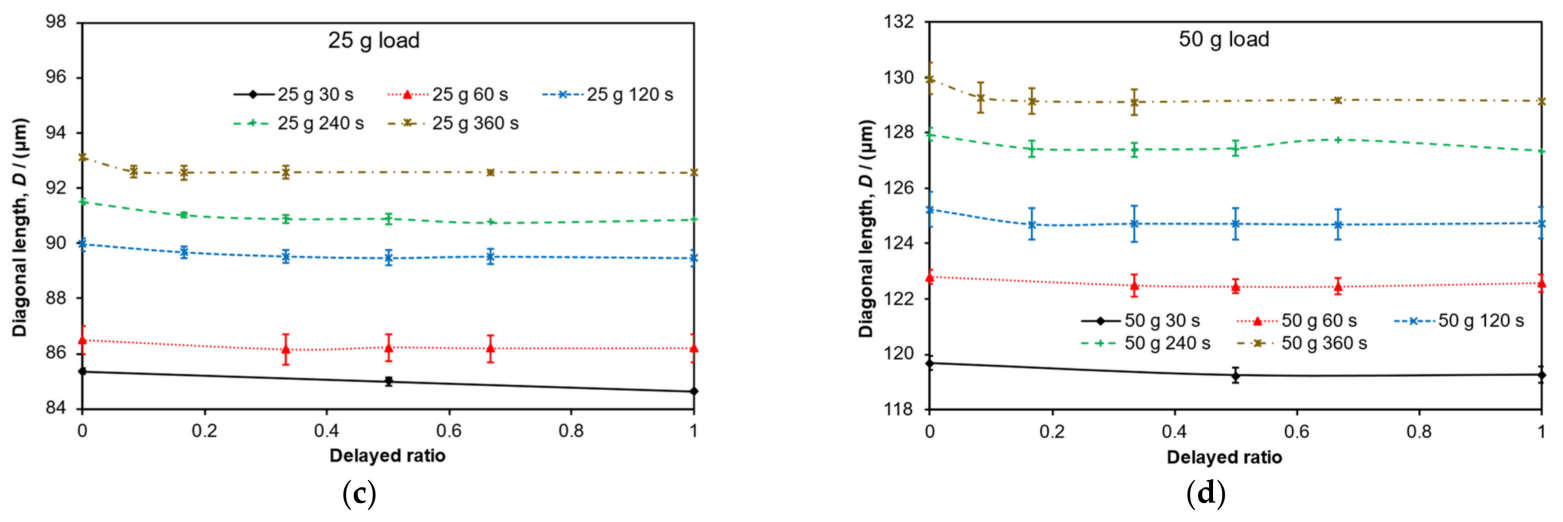
Relationship between the recovery of the diagonal length versus delayed ratio: Loads levels of (**a**) 5 g, (**b**) 10 g, (**c**) 25 g and (**d**) 50 g are shown with the corresponding dwell times. Error bars represent standard errors. Trendlines connecting the data points are shown.

Figure 4 shows the time series data for the recorded diagonal lengths. The figures show lengths versus delayed time ratio (the observation time relative to the dwell time) for all four load levels: (**a**) 5 g, (**b**) 10 g, (**c**) 25 g and (**d**) 50 g. The recorded curves show a minimal level of strain recovery (less than 1%) up to the delayed time of unity with little or no observable change thereafter. All indents were measured again 24 h later, and no significant changes had occurred over this time period compared to the measurements taken at a delayed time ratio of one. Given this information, all other indentation data were recorded at delayed ratios greater than unity but within 24 h.

### 3.4. Loads and Dwell Times

**Figure 5:**
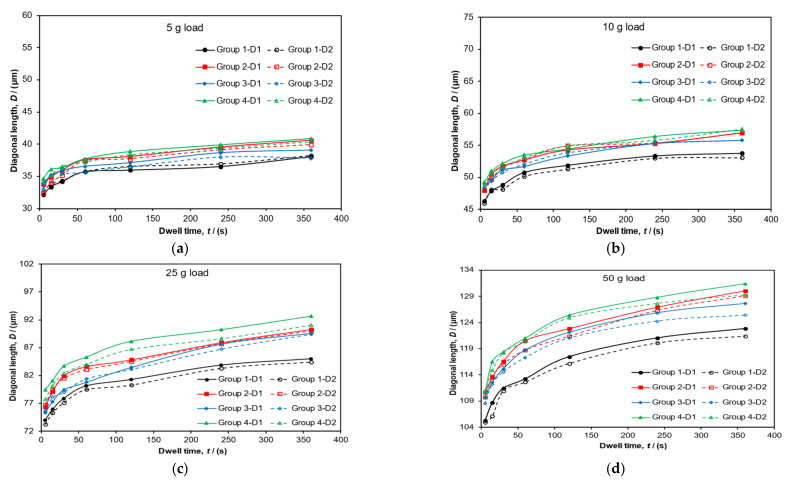
The relationship between diagonal lengths and dwell time at each load level. Trendlines connecting the data points are shown.

Figure 5 shows the results of all diagonal lengths measured in groups. Each group represents a batch of indents laid down in series during a single sitting but with different dwell times applied. Each group was completed on different days, and therefore, they should be considered as replicated runs of the same testing sequence. The numerical suffix added to the end of the group data represents individual diagonal directions. The suffix -D1 represents the horizontally orientated diagonal and -D2 represents the vertically orientated diagonal as it was viewed on screen by the operator at that time. Data were gathered at each load level of interest: (**a**) 5 g, (**b**) 10 g, (**c**) 25 g and (**d**) 50 g. Generally, for a fixed load level, the diagonal lengths grew bigger as the dwell time Increased, but this effect tended to plateau at longer dwell times greater than 350 s. Indent size increased with load level as was expected.

**Figure 6:**
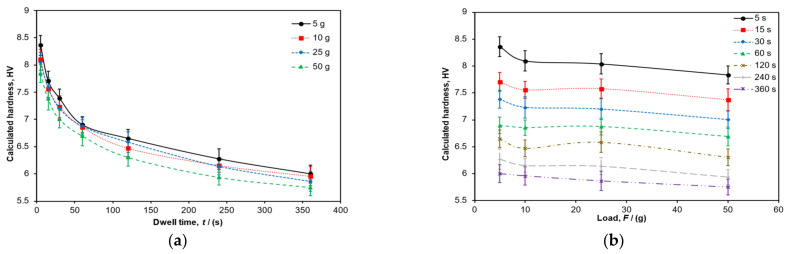
(**a**) Relationship between hardness value and dwell time for four load levels and (**b**) relationship between hardness value and load for seven dwell times. Trendlines connecting the data points are shown.

After applying Equation (1), the calculated Vickers hardness, *HV*, could be plotted against dwell time and load level. Figure 6a shows the calculated hardness data using the empirical diagonal measurements versus dwell time at each load level. As dwell time increased, the recovered diagonal sizes became larger, and the hardness number decreased, accordingly. This trend shows the importance of establishing a consistent parameter when acquiring material hardness data from polymers. Figure 6b shows the same data but in a different way—calculated hardness is plotted against load level. Again, as the diagonal measurements increase with load, the calculated hardness numbers decrease, but it can be seen that the hardness data are less sensitive to the changes in load level compared to dwell time.

### 3.5. Data Fitting Exercise

**Figure 7:**
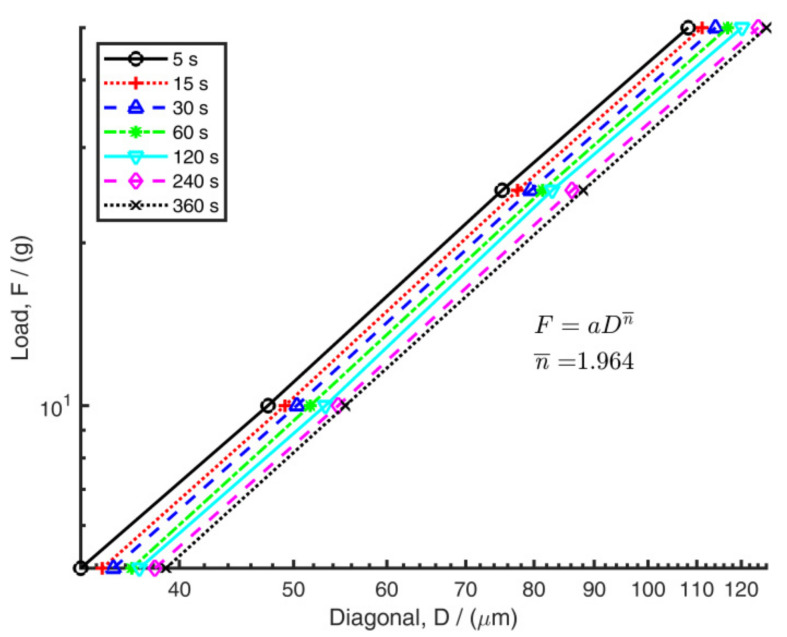
Relationship between diagonal length and load for seven dwell times. Lines of best fit are shown.

Figure 7 shows load versus indent diagonal plotted on a log–log scale. Trendlines represent the different dwell times. As expected, the plots show straight lines that are closely parallel. The average slope from all trendlines gave an index value of $\bar{n}=1.964$.

**Figure 8:**
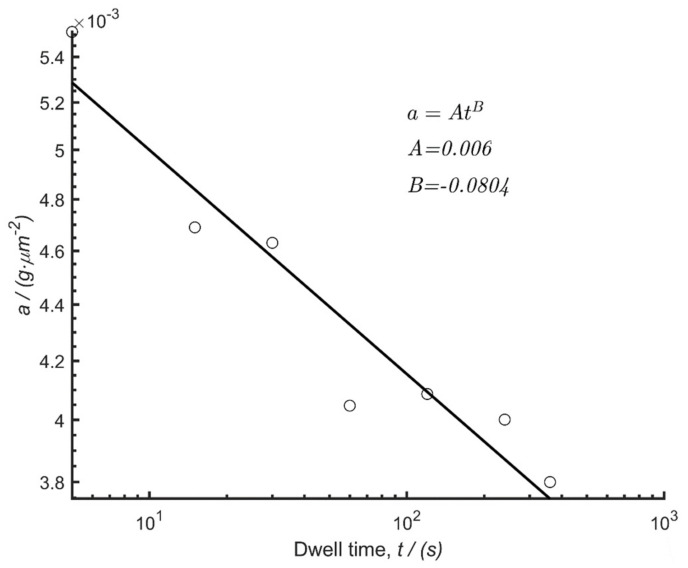
Relationship between parameter *a* and dwell time with a regression fit to get parameters *A* and *B*.

Since the *y*-axis intercepts of all the trendlines in Figure 7 are different, it was required to plot the parameter *a* from Equation (3) versus dwell time (Figure 8). On a semi-log scale, the trend is linear with a negative slope as expected. The fitting parameters, *A* and *B*, from Equation (4) were deduced from a regression line fit to give *A* = 0.006 and *B* = −0.0804.

**Figure 9:**
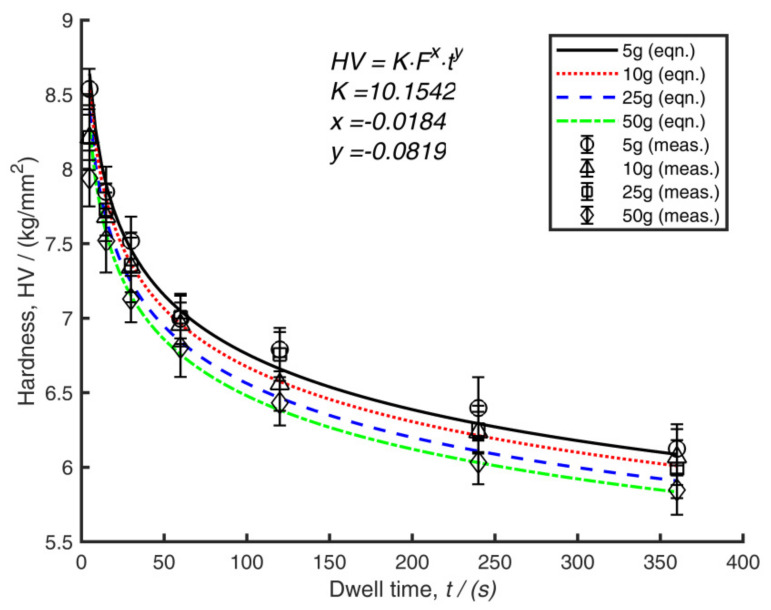
Comparison of the measured and predicted hardness versus dwell time over all four load levels. Lines of best fit are shown.

Finally, the fitting data were applied through Equation (5) to get values for the constants in Equation (6). Figure 9 shows the analytical hardness values (developed through the equation) against the measured hardness data (achieved through diagonal measurements). The prediction is seen as optimal with the following fitting parameters applied *K* = 10.1542, *x* = −0.0184, and *y* = −0.0819 (note that load is given in Equation (6) as grams).

### 3.6. Carbon Black Addition

Selecting the following parameter set, 5 g load, 60 s dwell time, and delayed ratio greater than 1, a series of tests were performed on samples with increasing levels of carbon black (0.5, 1, 2 and 3 wt.%). Overall, the carbon black is shown to have increased the hardness measurements with a non-linear increasing trend rate over the range investigated. The data are presented in comparison with the data from the literature (Koszkul [17])

## 4. Discussion

As Crawford [3] rightly points out, the term hardness is somewhat ‘indefinite’, and a hardness number cannot be considered as a fundamental property of matter. Because the term hardness only takes on significant meaning when the precise test method is established, hardness numbers need to be used with due care. Hardness is typically a function of other material properties, such as modulus, yield strength, Poisson’s ratio, etc. However, as shown in this analysis, hardness values for polymers are also highly dependent on the parameter set chosen. It was shown that the calculated hardness reduced as the dwell time increased (Figure 9), but this did not necessarily mean that the material became softer due to the action of the longer dwell times; instead, the viscoelastic response of the material affected the hardness reading due to longer indentation application timings. Therefore, it has been shown that the specific test parameters, namely, load and dwell time need to be considered carefully and quoted when providing the hardness numbers.

As shown by Crawford [3], a fitting curve in the form of Equation (5) can be applied to predict the response of the hardness values to loads and dwell times. Table 1 compares the values for iPP that were discovered in this research versus the values provided by Crawford. It can be seen that the parameters derived through the current analysis differ significantly from those provided in the literature. It is expected that the fitting parameters presented for each case depend on the condition of the material. Therefore, it should be expected that the information presented in Table 1 is dependent on the crystallinity or microstructure of that particular sample. Therefore, again, caution should be exercised when gathering data across different samples. Since, in this case, all data in Figure 9 were taken from samples processed under the same conditions (i.e., the sample is the same), it is safe to assume that the crystallinity was consistent in the current case.

The crystallinity is estimated by rearranging the additivity law (Equation (1)) to give:

(7) $$\alpha =\frac{HV-{\;H}_{A}}{H_{C}-H_{A}}.$$

In order to use this law, the hardness values have to be measured using consistent hardness test parameters. Martinez Salazar et al. [15] measured hardness over 6 s using two loads: 0.25 and 0.5 N. Using this input information along with data from Table 1, the estimated hardness is 81 MPa at a load of 0.25 N and is 80 MPa at a load of 0.5 N (i.e., assuming a dwell time of 6 s). Based on the data for *H*_C_ and *H*_A_ from Reference [15], the crystallinity is therefore estimated to be within the range of 58% to 59%. The narrow range demonstrated in this estimated result (1% difference after a doubling of load parameter) is due to the low load dependency of hardness in this test range (as clearly shown in Figure 6b). The average crystallinity of the sample was confirmed using Differential Scanning Calorimetry (DSC) to be 36.44%. Hence, the estimation approach used here is an overestimate of the crystallinity measured directly by an accepted method. Crystallinity gradients are known to occur in rapidly cooled, relatively thick samples due to differences in cooling rate across the sample. The hardness measurements were all taken along the centreline of the sample, where the cooling rate is expected to be the lowest. Lower cooling rates would lead to higher crystallinity in the centre of the sample.

**Figure 10:**
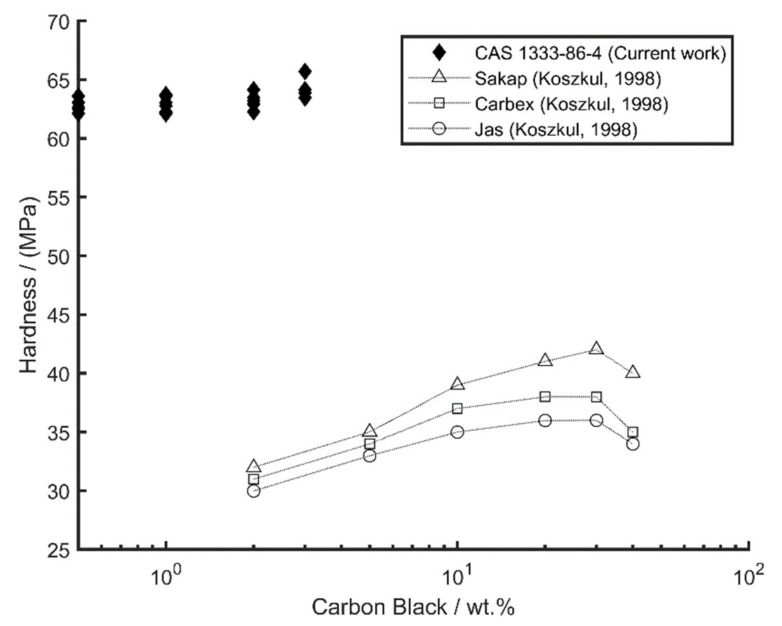
Hardness of iPP with addition of CB. Current data show up to a 3 wt.% of CAS 1333-86-4. Data from the literature showed up to 40 wt.% with trendlines. Different tradenames of CB are provided in [17].

The application of hardness testing to iPP with CB additions was presented in Figure 10. Carbon black addition levels around 3 wt.% are technically useful in transmission laser welding applications [20] and, with a percolation limit of 0.97 wt.%, are also used for increasing the electrical conductivity [21] and electrical shielding capability [22]. Comparisons are made between our data and data from the literature [17].

Koszkul used a Knoop indenter load of 132.4 N but did not give a dwell time. Crystallinity percentages were cited from 29.3% to 47.4% using a DSC measurement method (or from 58.3% to 64.7% when measured using the Wide-angle Scattered X-radiation (WAXS) method), whereas in our case, crystallinity was measured using DSC and found to be in the range from 37.28% to 41.2%. The crystallinity values are similar, but since hardness is significantly different, these results highlight the pitfall of comparing data across different literature sources when test parameters are not fully known. As mentioned, we do not know the dwell time for the data in [17] and cannot compare these two datasets with confidence. Both data sets (the current data and the data from the literature) show an increasing trend in hardness with CB addition (even over the range of 3 wt.% of the current data), but the current hardness data are approximately double the values cited in the literature. It is likely to be the case, as speculated in Flores et al. [16], that Koszkul used long dwell times for hardness testing, which would give lower hardness values. It should also be noted that the load levels used in Koszkul are orders of magnitude higher than those used here. The higher load levels (orders of magnitude higher) would tend to give lower hardness values as demonstrated in Equation (6).

In overview, loads applied throughout this analysis are lower than those used by Crawford and others. The practical advantage of the current regime is that indents can be measured ‘down the eyepiece’ of most commercially available microhardness testers. The analysis shows the importance of proceeding with a calibration exercise before attempting to interpret micro Vickers hardness numbers.

## 5. Conclusions

The aim of this study was to determine the suitability of micro Vickers hardness testing on iPP and to answer the question of what empirical and mathematical framework, if any, must be adopted to allow for comparisons to be made between different hardness datasets obtained under different test parameters. We demonstrated that the framework provided by Crawford [3] can be used to transpose hardness datasets obtained under a known set of test conditions (load and dwell time) to another set of test conditions. However, it is imperative that the parameters in Crawford’s equation (Equation (6)) be developed empirically on a case-by-case basis. Several ancillary tasks became apparent as the requirement for low loading meant that the smaller indents placed greater emphasis on sample preparation. Particular focus was placed on reviewing suitable cold casting compounds and on maintaining the temperature below a threshold of 25 °C. Epoxy resins that cure at room temperature were deemed most suitable for this application but with the disadvantage that curing times are much longer than an alternative acrylic compound. The acrylic gave a peak temperature of around 70 °C, which was deemed unacceptable.

For surface preparation, it was shown that wet grinding, using graduated levels of silicon carbide papers up to P2500 followed with a changeover to graduated levels of alumina suspension polishing and finishing with 0.05 µm suspension, is recommended.

The importance of calibrating the load versus the dwell time was discussed at length. A data fitting analysis was performed, and it was shown that the fitting parameters discovered were significantly different from those reported in the literature [3] emphasising the importance of operator calibration before applying the test method. In addition, for the reasons just explained, caution should be exercised in comparing with datasets from the literature that are calibrated under different or unknown circumstances. Demonstrative examples are provided to show the applicability of hardness testing and the pitfalls associated when dwell time is not cited with the hardness test method.

## Figures and Tables

**Table 1:** Fitting parameters for Equation (6).

Fitting Parameter	Current Investigation	Crawford [3]
*K*	10.1542	15.34
*x*	−0.0184	−0.104
*y*	−0.0819	−0.0618

## Data Availability

Not applicable.
